# Compounds from *Dryopteris Fragrans* (L.) Schott with Cytotoxic Activity

**DOI:** 10.3390/molecules19033345

**Published:** 2014-03-18

**Authors:** Dan-Dan Zhao, Qin-Shi Zhao, Li Liu, Zhong-Qin Chen, Wei-Min Zeng, Hong Lei, Yan-Long Zhang

**Affiliations:** 1 Key Laboratory of Molecular Biology of Heilongjiang Province, College of Life Science, Heilongjiang University, Harbin 150080, China; E-Mails: zhaodandan@hlju.edu.cn (D.-D.Z.); liliuhlju@gmail.com (L.L.); zqchenhd@163.com (Z.-Q.C.); wmzenghd@163.com (W.-M.Z.); hleihd@163.com (H.L.); 2 State Key Laboratory of Phytochemistry and Plant Resources in West China, Kunming Institute of Botany, Chinese Academy of Sciences, Kunming 650204, China; E-Mail: qinshizhao@mail.kib.ac.cn

**Keywords:** *Dryopteris fragrans* (L.) Schott, compounds, MTT, cytotoxic activity

## Abstract

One new coumarin, dryofracoumarin A (**1**), and eight known compounds **2**–**9** were isolated from *Dryopteris fragrans* (L.) Schott. Their structures were established on the basis of extensive spectroscopic data analyses and comparison with reported spectroscopic data. The new compound **1** was determined to be 8-hydroxyl-4-isopropyl-7-methyl-6-methyl-2*H*-benzopyran-2-one. Two dimers, *trans*- and *cis*-3-(3,4-dimethoxyphen-yl)-4-[(*E*)-3,4-dimethoxystyryl]cyclohex-1-ene (compounds **8** and 9), were isolated from the *Dryopteris* genus for the first time. The other six were esculetin (**2**), isoscopoletin (**3**), methylphlorbutyrophenone (**4**), aspidinol (**5**), albicanol (**6**) and (*E*)-4-(3,4-dimethoxyphen-yl)but-3-en-1-ol (**7**). All compounds were evaluated for their cytotoxic effects by the MTT assay. Compounds **2**, **3**, **8** and **9** showed significantly cytotoxic effects against three cell lines (A549, MCF7 and HepG2), **1** and **5** against two cell lines (A549 and MCF7), and **6** against one cell line (MCF7). Their IC_50_ values ranged between 2.73 ± 0.86 μM and 24.14 ± 3.12 μM. These active compounds might be promising lead compounds for the treatment of cancer.

## 1. Introduction

Traditional Chinese Medicines (TCMs), are commonly used as representative alternative and complementary medicines for the treatment of cancer in Asian countries [1]. The current treatment modality for patients with lung, breast or other cancer is chemotherapy based on taxane, a famous and efficient drug isolated from the bark of the Pacific yew tree [2,3,4]. However, its efficacy is frequently attenuated due to drug resistance and side effects [5], so it is urgent to identify new targets to treat cancer from TCMs.

**Figure 1:**
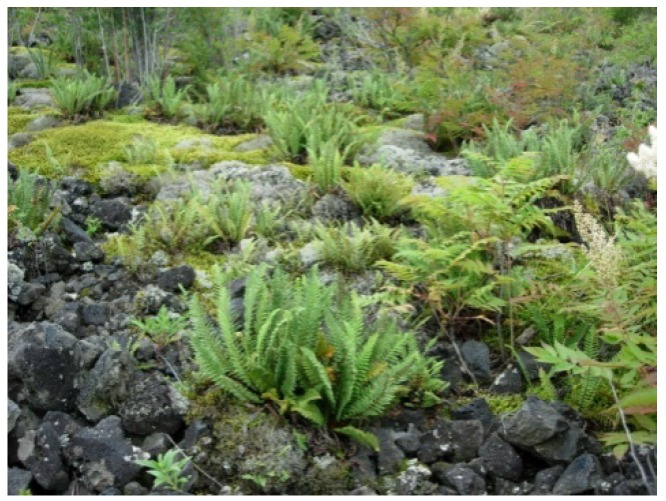
*D. fragrans* in the lava cracks of the Wu-Da-Lian-Chi volcanoes.

*Dryopteris fragrans* (L.) Schott (Chinese name: Xiang-Lin-Mao-Jue) is a deciduous perennial herb of the *Dryopteris* genus, which grows mainly in small communities on the talc slopes of the alpine region and the lava cracks of volcanoes (Figure 1). It is distributed in many countries, especially in Wu-Da-Lian-Chi, Heilongjiang Province, Northeast China [6]. *D. fragrans* was used for the treatment of skin diseases, especially on treatment of psoriasis, arthritis, rash, dermatitis and barbiers [7,8]. Previous investigations also showed that its extracts have antimicrobial properties and anticancer activity [9,10]. 

Many constituents from *D. fragrans* have been isolated, including sesquiterpenes, phloroglucins, phenolic glycosides, itosterols and essential oils. Among them, more than 10 compounds from have anticancer activities [11,12,13,14,15]. For example, aspidin BB and albicanol suppressed *in vivo* two-stage carcinogenesis on mouse skin [10]. However, there are no reports available on the cytotoxic activity of compounds from the ethanolic extract of *D. fragrans*. In this study, compounds with significant cytotoxic activity on A549, MCF7 and HepG2 cells (*in vitro* lung, breast and liver cancer models) were reported.

## 2. Results and Discussion

### 2.1. Compounds from D. fragrans

**Figure 2:**
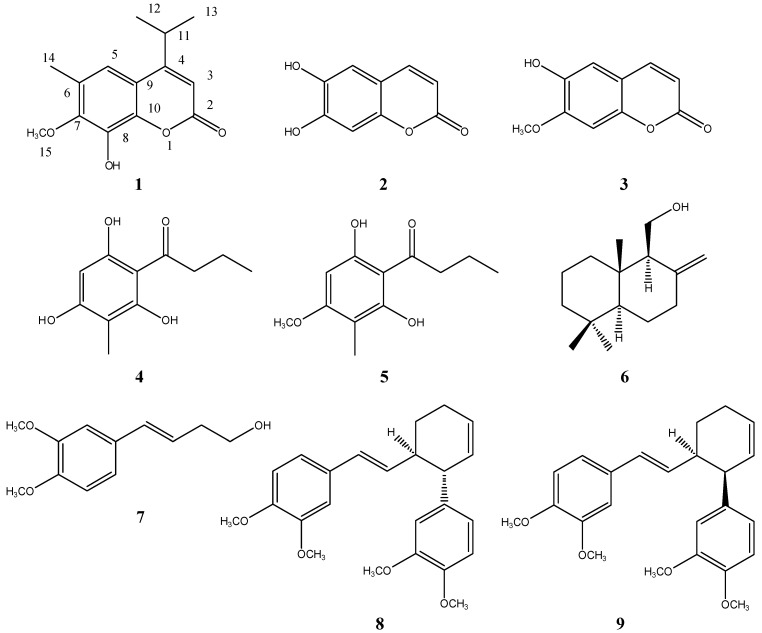
Isolated compounds (**1**–**9**) from *D. fragrans*.

One new coumarin compound **1** was isolated from the 95% ethanol extract of *D. fragrans*, together with eight known compounds. They are 8-hydroxyl-4-iso-propyl-7-methyl-6-methyl-2*H*-benzopyran-2-one (**1**, named as dryofracoumarin A), 6,7-hihydroxycoumarin (**2**, esculetin), 6-hydroxy-7-methoxy-2H-1-benzopyran-2-one (**3**, isoscopoletin), methylphlorbutyrophenone (**4**), 1-(2,4,6-trihydroxy-3-methylphenyl)-1-butanone (**5**, aspidinol), 2,4-dihydroxy-6-methoxyl-3,5-dimethylacetophenone (**6**, albicanol), (*E*)-4-(3,4-dimethoxyphenyl)but-3-en-1-ol (**7**), *trans*-3-(3,4-dimethoxyphenyl)-4-[(*E*)-3,4-dimethoxystyryl]cyclohex-1-ene (**8**) and *cis*-3-(3,4-dimethoxyphenyl)-4-[(*E*)-3,4-dimethoxystyryl] cyclohex-1-ene (**9**). Their structures are as shown in Figure 2.

### 2.2. Chemical Structure Identification and Spectroscopic Data

Compound **1** was obtained as colorless crystals. ESIMS analysis produced a pseudomolecular ion at *m/z* 249 [M+H]^+^, and positive HR-ESI-MS gave a molecular formula of C_14_H_16_O_4_ from the ion at *m/z* 249.1133 [M+H]^+^ (calcd. for C_14_H_17_O_4_, 249.1126), with seven degrees of unsaturation. The IR spectrum showed the presence of hydroxyl group (3,357 cm^−1^) and lactone carbonyl (1,699 cm^−1^) functions. The general appearance of its ^1^H- and ^13^C-NMR spectra (Table 1), in addition to the information obtained from its mass spectrum, suggested a coumarin skeleton for compound **1** [16].

By analysis of the ^1^H-NMR spectroscopic data of compound **1** four methyls, two belonging to an isopropyl group *δ*_H_ 1.25 (d, *J* = 6.9 Hz, 3H, H-12); *δ*_H_ 1.25 (d, *J* = 6.9Hz, 3H, H-13); one aromatic methyl *δ*_H_ 2.26 (s, 3H, H-14); one methoxyl group *δ*_H_ 3.89 (s, 3H, H-15); and three methines *δ*_H_ 6.15 (s, 1H, H-3) (an olefinic proton), 6.95 (s, 1H, H-5) (aromatic proton), and another methine belonging to an isopropyl group *δ*_H_ 3.24 (m, 1H, H-11) were figured out. The ^13^C-NMR and DEPT experiment revealed the presence of fifteen carbon resonances, comprising four methyls, one methoxyl, three methines (including one olefinic) and seven quaternary carbons (including one carbonyl).

**Table 1:** ^1^H- and ^13^C-NMR data of compound **1**.

No.	*δ*_C_^a^	*δ*_H_^b^ (*J* in Hz)	No.	*δ*_C_^a^	*δ*_H_^b^ (*J* in Hz)
1			9	116.1 (C)	
2	163.7 (C)		10	143.5 (C)	
3	109.6 (CH)	6.15 (1H, s)	11	29.8 (CH)	3.24 (1H, m)
4	165.4 (C)		12	22.3 (CH_3_)	1.25 (3H, d, 6.8)
5	116.3 (CH)	6.95 (1H, s)	13	22.3 (CH_3_)	1.25 (3H, d, 6.8)
6	129.0 (C)		14	16.3 (CH_3_)	2.26 (3H, s)
7	150.4 (C)		15	60.7 (OCH_3_)	3.89 (3H, s)
8	139.1 (C)				

^a & b^ Recorded at 400 MHz; *J* in Hz within parentheses.

**Figure 3:**
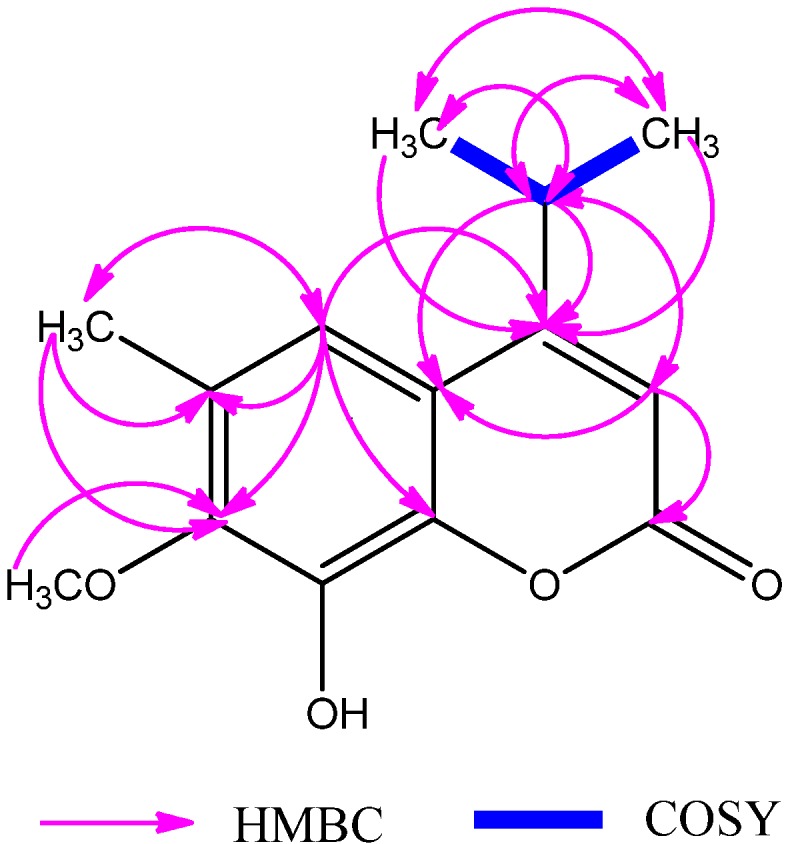
Key HMBC and COSY correlations of compound **1**.

In the HMBC and COSY correlation spectra of compound **1** (Figure 3), long-range correlations from H-12, 13 (*δ*_H_ 1.25, 1.25) to C-4 (*δ*_C_ 165.4), and the long-range correlations H-11 (*δ*_H_ 3.24) to C-3, C-4, C-9 (*δ*_C_ 109.6, 165.4, 116.1), suggested that the isopropyl was fused to C-4. Long-range correlations from H-14 (*δ*_H_ 2.26) to C-5, C-6, C-7 (*δ*_C_ 116.3, 129.0, 150.4) confirmed that the isolated methyl was located at C-6. Another long-range correlation from H-15 (*δ*_H_ 3.89) to C-7 (*δ*_C_ 150.4) indicated that the methoxyl group was linked to C-7. Based on the spectroscopic data analyses, the structure of **1** was determined to be 8-hydroxy-4-isopropyl-7-methoxy-6-methyl-2*H*-chromen-2-one and this new compounds was named dryofracoumarin A.

### 2.3. Effects of Compounds on Cytotoxic Activity

Lung cancer in males or breast cancer in females are the most frequently diagnosed cancers and the leading cause of cancer death in both developed and developing countries [17,18]. All compounds were investigated for their anticancer activity *in vitro* against A549 (human lung cancer), MCF7 (human breast cancer), along with HepG2 [19] (human liver cancer) cancer cell lines using pseudolaric acid B as standard [20]. Pseudolaric acid B was isolated from the ethanolic extract of the root bark of *Pseudolarix kaempferi* with the purity of 99.3% by Dr. Qinshi Zhao (Kunming Institute of Botany, Chinese Academy of Sciences). IC_50_ values, the concentration of the test compounds inhibiting 50% of the cell growth at 48 h, was calculated by Reed and Muench’s method [21]. Among all the compounds, **2**, **3**, **8** and **9** showed promising activity against three cancer cell lines at below 30 μM concentration, with **1** and **5** against two lines (A549 and MCF7) and **6** against one line (MCF7) (Table 2).

**Table 2:** *In vitro* cytotoxicity of compounds against three cancer cell lines.

Compound No.	IC_50_^a^ ± SE (μM)			
	A549	MCF7	HepG2	
**1**	6.56 ± 1.59	10.14 ± 1.85	– ^b^	
**2**	3.82 ± 0.23	2.73 ± 0.86	10.15 ± 1.77	
**3**	5.25 ± 1.62	8.58 ± 1.34	4.76 ± 1.01	
**4**	–	–	–	
**5**	12.59 ± 2.74	10.58 ± 1.56	–	
**6**	–	24.14 ± 3.12	–	
**7**	–	–	–	
**8**	14.13 ± 3.72	17.81 ± 4.11	17.90 ± 5.21	
**9**	17.25 ± 3.79	16.45 ± 5.80	23.75 ± 4.57	
**Pseudolaric acid B** ^c^	2.81 ± 0.45	2.44 ± 0.33	1.50 ± 0.28	

^a^ IC_50_ values represent the mean ± SE of three individual observations; ^b^ “–” indicated that the compound was not active below 30 μM concentration; ^c^ Pseudolaric acid B was as positive control [20].

## 3. Experimental

### 3.1. General

Melting points were obtained on an XRC-1 micro melting point apparatus. Optical rotations were measured on a JASCO DIP-370 digital polarimeter. IR spectra were obtained on a Bruker Tensor 27 spectrometer with KBr pellets. UV spectra were recorded using a Shimadzu UV-210A spectrophoto-meter. ESIMS (including HRESIMS) were carried out on API QSTAR Pulsar I (Applied Biosystems, MDS Sciex, Framingham, MA, USA) and VG Autospec-3000 mass spectrometers (AB SCIEX mass spectrometers). 1D and 2D NMR spectra were performed on AM-400 spectrometers (Bruker Corporation, Fällanden*,* Switzerland) with TMS as an internal standard. Column chromatography was performed on silica gel (SiO_2_: 200–300 and 100–200 mesh, Qingdao Marine Chemical Ltd., Qingdao, China), MCI gel (75–150 μm; Mitsubishi Chemical Corporation, Tokyo, Japan), Sephadex LH-20 (Amersham Pharmacia Biotech, Stockholm, Sweden). Semi-preparative HPLC was performed on an Agilent 1100 liquid chromatograph equipped with a Zorbax SB-C18 (9.4 mm × 25 cm) column. Fractions were monitored using TLC, and spots were visualized by heating silica gel plates (G254, Qingdao Marine Chemical Ltd) immersed with 10% H_2_SO_4_ in ethanol. All solvents were distilled before use.

### 3.2. Plant Material

*Dryopteris fragrans* (L.) Schott was collected in Wu-Da-Lian-Chi, Heilongjiang Province, China, in August 2009 (Figure 1), and identified by Prof. Zhen-Yue Wang (Z.-Y.W., Heilongjiang University of Chinese Medicine). The voucher specimen (Registration number: XLMJ-20110812) of this plant was deposited in the Herbarium of Heilongjiang University of Chinese Medicine, Harbin, China.

### 3.3. Extraction and Isolation

Air-dried, powdered whole plants of *Dryopteris fragrans* (L.) Schott (3 kg) were extracted three times with 95% ethanol at room temperature. After removal of the solvent by evaporation, the residue (240 g) was suspended in H_2_O and partitioned with EtOAc. The EtOAc fraction (135 g) was subjected to silica gel column chromatography with a gradient elution system of petroleum ether–acetone (90:10–0:100, *v/v*) to obtain five fractions (Fr I–Fr V). Fr I was separated into four subfractions (Fr I 1–4) by MCI (MeOH/H_2_O, 30:70–100:0, *v/v*) and gave the crude crystals of compound **2** (>99% HPLC). Fr I 2 was further subjected to silica gel chromatography with gradient mixture of CHCl_3_ and MeOH to obtain three sub-fractions (Fr I 2-1, Fr I 2-2 and Fr I 2-3). Sub-fraction Fr I 2-2 was purified by HPLC (MeOH/H_2_O, 55:45, *v/v*) to yield compound 4 (>97% HPLC) and compound **1** (>99% HPLC). Fr I 2-1 and Fr I 2-3 were purified using Sephadex LH-20 (CHCl_3_/MeOH, 1:1, *v/v*) to obtain compound **7** (>97% HPLC). Fr II was decolorized with MCI (MeOH/H_2_O, 10:90–100:0, *v/v*) to yield three sub-fractions (Fr II 1, Fr II 2 and Fr II 3). Fr II 1 was further separated by silica gel column chromatography with gradient mixture of petroleum ether and Me_2_CO (80:20; 70:30; 60:40, *v/v*) to give Fr II 1-1, Fr II 1-2 and Fr II 1-3. Fr II 1-1 was further purified by subjected to Sephadex LH-20 column chromatography (MeOH) to yield compound **6** (>97% HPLC). Fr II 1-2 was purified by semi-preparative HPLC (MeOH/H_2_O, 55:45, eluting for 20 min with a flow rate of 30 mL/min) to afford compound **8** (>97% HPLC) and compound **9** (>98% HPLC). Fr II 1-3 was further purified by subjected to Sephadex LH-20 column chromatography (MeOH) to give compound **5** (>98% HPLC) and compound **3** (>99% HPLC).

### 3.4. Characterization of Isolated Compounds

*Dryofracoumarin A* (**1**). Colorless crystals, Mp: 69.5 °C, C_14_H_16_O_4_, positive ESIMS: *m/z* 271 [M+Na]^+^; positive HRESIMS [M+Na]^+^ *m/z* 271.1133 (calcd. for C_14_H_16_O_4_ Na,248.1133), UV (CD_3_OD) *λ*_max_ (log *ε*): 303 nm; IR (KBr) *ν*_max_ 3357, 2965, 1611, 1570, 1442, 1699 cm^−1^, ^1^H and ^13^C data, see Table 1.

*Esculetin* (**2**). Yellow needles, Mp: 268–269 °C (lit [22] Mp: 268–270 °C), C_9_H_6_O_4_, ESI-MS *m/z*: 179 [M+H] ^+^, ^1^H-NMR (MeOD): 6.17 (1H, d, *J* = 9.0 Hz, H-3), 7.78 (1H, d, *J* = 9.0 Hz, H-4), 6.92 (1H, s, H-5), 6.74 (1H, brs, H-8). ^13^C-NMR (MeOD): 164.2 (C-2), 112.7 (C-3), 146.0 (C-4), 113.0 (C-5), 144.7 (C-6), 150.6 (C-7), 103.6 (C-8), 152.1 (C-9), 112.5 (C-10). 

*Isoscopoletin* (**3**). Light yellow needles, Mp: 138–140 °C (lit [23] Mp: 138–140 °C), C_10_H_8_O_4_ ESI-MS *m/z*: 193 [M+H]^+^, ^1^H-NMR (CDCl_3_): 6.29 (1H, d, *J* = 9.6 Hz, H-3), 7.62 (1H, d, *J* = 9.6 Hz, H-4), 6.87 (1H, brs, H-5), 6.94 (1H, brs, H-8), 6.22 (1H, brs, -OH), 3.97 (3H, s, -OCH_3_). ^13^C-NMR (CDCl_3_): 161.5 (C-2), 111.5 (C-3), 143.3 (C-4), 107.4 (C-5), 149.7 (C-6), 144.0 (C-7), 103.1 (C-8), 150.2 (C-9), 113.4 (C-10), 56.4 (OCH_3_).

*Methyl-phlor-butyrophen-on* (**4**). Yellow needles, Mp: 166 °C (lit [24] Mp: 166–167 °C), C_11_H_14_O_4_, ESI-MS *m/z* 233 [M+Na]^+^, ^1^H-NMR (CD_3_OD): 0.96 (3H, m, H-4), 1.67 (2H, m, H-3), 1.90 (3H, s, H-5), 3.01 (2H, m, H-2), 4.61 (1H, s, H-3'), 5.86 (1H, s, -OH); ^13^C-NMR (CD_3_OD): 7.3 (C-5), 14.4 (C-4), 19.6 (C-3), 46.9 (C-2), 94.7 (C-3'), 103.5 (C-1'), 105.1(C-5'), 161.3 (C-2'), 163.8 (C-4'), 163.8 (C-6'), 207.4 (C-1). 

*Aspidinol* (**5**). Yellow needles, Mp: 144–145°C (lit [25] Mp: 144–145 °C), C_12_H_16_O_4_, ESI-MS *m/z* 247 [M+Na]^+^, ^1^H-NMR (CD_3_OD): 0.85 (3H, t, *J* = 7 Hz, H-4), 1.56 (2H, m, H-3), 1.83 (3H, s, H-5), 2.94 (2H, t, *J* = 7 Hz, H-2), 3.68 (3H, s, OMe), 4.02 (1H, s, H-4'); ^13^C-NMR (CD_3_OD): 6.7 (C-5), 13. 7 (C-4), 18.1 (C-3), 45.9 (C-2), 55.0 (OMe), 90.0 (C-5'), 103.6 (C-1'), 104.6 (C-3'), 160.4 (C-6'), 161.9 (C-2'), 163.4 (C-4'), 206. 9 (C-1). 

*Albicanol* (**6**). Colorless crystals, Mp: 68–70 °C (lit [10] Mp: 68–69 °C), C_15_H_26_O, ESI-MS *m/z* 247 [M+Na]^+^, [α]_D_^25^ = +21.2 (c 0.015, EtOH), ^1^H-NMR (CD_3_OD): 0.89 (3H, s, Me-14), 0.83 (3H, s, Me-13), 0.73 (3H, s, Me-12), 1.17 (1H, dd, *J* = 12.7, 2.6 Hz H-5), 1.14 (1H, m, H-1), 1.23 (1H, m, H-3), 1.33 (1H, m, H-6), 1.43 (1H, m, H-3), 1.40 (1H, m, H-2), 1.59 (1H, m, H-2), 1.40 (1H, m, H-1), 1.63 (1H, brd, J = 14.0 Hz, H-6), 2.40 (1H, m, H-9), 1.87 (1H, brs, H-16), 1.75 (1H, m, H-7), 1.89 (1H, brd, *J* = 12.8 Hz, H-7), 3.69 (1H, dd, *J* = 10.8, 10.8 Hz, H-11), 3.82 (1H, dd, *J* = 10.8, 3.6 Hz, H-11), 4.66 (1H, s, H-15), 4.90 (1H, s, H-15). ^13^C-NMR (CD_3_OD): 15.7 (C-14), 29.3 (C-2), 22.3 (C-13), 15.7 (C-14), 25.3 (C-6), 34.2 (C-4), 34.4 (C-12), 39.0 (C-7), 39.9 (C-10), 40.3 (C-10), 43.2 (C-3), 56.6 (C-5), 58.9 (C-11), 59.8 (C-9), 107.7 (C-15), 148.4 (C-8).

*(E)-4-(3,4-dimethoxyphenyl)but-3-en-1-ol* (**7**). Light yellow oil, C_12_H_16_O_3_, ESI-MS *m/z* 231 [M+Na]^+^, ^1^H-NMR (CD_3_OD):2.39 (2H, q, *J* = 6.6 Hz, H-2), 3.64 (2H, t, * J* = 6.6 Hz, H-1), 6.15 (1H, d, * J* = 15.8 Hz, H-3), 3.79 (3H, s, -OMe), 3.82 (3H, s, -OMe), 6.40 (1H, d, *J* = 15.8 Hz, H-4), 6.84 (1H, d, *J* = 8.5 Hz, H-5'), 6.88 (1H, d, *J* = 8.5 Hz, H-4'), 6.99 (1H, s, H-1'). ^13^C-NMR (CD_3_OD): 37.4(C-2), 56.4(-OMe), 56.4 (-OMe), 62.9 (C-1), 110.3 (C-2'), 112.9 (C-5'), 120.3 (C-6'), 126.0 (C-3), 132.6 (C-1'), 132.7 (C-4), 149.8 (C-4'), 150.5 (C-3') [26,27].

*Trans-3-(3, 4-dimethoxyphenyl)-4-[(E)-3,4-dimethoxystyryl] cyclohex-1-ene* (**8**). Colorless needles, Mp: 99.5 °C (lit [28,29] Mp: 99.5–100 °C), C_24_H_28_O_4_, ESI-MS *m/z* 403 [M+Na]^+^, ^1^H-NMR (CD_3_OD): 1.66 (1H, m, H-5), 1.85 (1H, m, H-5), 2.18 (1H, m, H-6), 2.37 (1H, m, H-6), 2.86 (1H, m, H-4), 3.20 (1H, m, H-3), 5.60 (1H, dd, *J* = 10.0, 3.0 Hz, H-2), 5.63 (1H, dd, *J* = 10.0, 3.0 Hz, H-1), 6.03 (1H, dd, *J* = 16.0, 7.0 Hz, H-1''), 6.70–6.81 (1H, s, H-2'), 6.09 (1H, d, *J* = 16.0 Hz, H-2''), 6.70–6.81 (1H, s, H-2'''), 6.70–6.81 (1H, m, H-5'), 6.70–6.81 (1H, s, H-5'''), 6.70–6.81 (1H, m, H-6'), 6.70–6.81 (1H, s, H-6'''), 3.72 (3H, s, -OMe), 3.73 (3H, s, -OMe), 3.75 (3H, s, -OMe), 3.77 (3H, s, -OMe); ^13^C-NMR (CD3OD): 25.2 (C-6), 29.8 (C-5), 46.5 (C-4), 48.7 (C-3), 55.9 (-OMe), 56.0 (-OMe), 56.0 (-OMe), 56.0 (-OMe), 110.2 (C-2'''), 112.5 (C-2'), 112.6 (C-5'), 113.3 (C-5'''), 119.6 (C-6'''), 121.0 (C-6'), 127.8 (C-2''), 129.9 (C-1), 131.4 (C-2), 131.8 (C-1'''), 132.9 (C-1''), 138.4 (C-1'), 148.7 (C-4'), 149.6 (C-4'''), 150.0 (C-3'), 150.3 (C-3''').

*Cis-3-(3, 4-dimethoxyphenyl)-4-[(E)-3,4-dimethoxystyryl] cyclohex-1-ene* (**9**). Colorless needles, Mp: 77.5 °C (lit [28,29] Mp: 78 °C), C_24_H_28_O_4_, ESI-MS *m/z* 403 [M+Na]^+^, ^1^H-NMR (CD_3_OD): 1.67 (1H, m, H-5), 2.04 (1H, m, H-5), 2.18 (1H, m, H-6), 2.23 (1H, m, H-6), 2.70 (1H, m, H-4), 3.53 (1H, m, H-3), 3.67 (3H, s, -OMe), 3.74 (3H, s, -OMe), 3.74 (3H, s, -OMe), 3.75 (3H, s, -OMe), 5.78 (1H, dt, *J* = 10.0, 2.0 Hz, H-2), 5.96 (1H, brd, *J* = 10.0 Hz, H-1), 5.95 (1H, dd, *J* = 16.0, 9.0 Hz, H-1''), 6.77 (1H, m, H-2'), 6.24 (1H, d, *J* = 16.0 Hz, H-2''), 6.86 (1H, s, H-2'''), 6.83 (1H, m, H-5'), 6.77 (1H, m, H-5'''), 6.75 (1H, m, H-6'), 6.70 (1H, d, H-6'''); ^13^C-NMR (CD3OD): 25.1 (C-5), 25.1 (C-6), 43.2 (C-4), 46.3 (C-3), 55.9(-OMe), 55.9 (-OMe), 56.0 (-OMe), 56.0 (-OMe), 110.1 (C-2'''), 111.9 (C-2'), 112.6 (C-5'), 115.1 (C-5'''), 119.6 (C-6'''), 122.6 (C-6'), 128.5 (C-1), 129.4 (C-2''), 130.7 (C-2), 132.0 (C-1'''), 132.5 (C-1''), 134.7 (C-1'), 148.9 (C-4'), 149.5 (C-4'''), 149.6 (C-3'), 150.3 (C-3''').

### 3.5. MTT Assay

Human A549, MCF7 and HepG2 cell lines were obtained from Cell Library of Committee on Type Culture Collection of Chinese Academy of Sciences. Cultures were maintained in 95% air and 5% CO_2_ at 37 °C in RPMI 1640 with 10% FBS, 2 mM L-glutamine, 100 U/mL penicillin and 100 U/mL streptomycin. Cytotoxic activity was measured by using MTT assay as described previously [30]. Absorbance was measured at 570 nm in a plate microreader (TECAN Infinite200, Eastwin Life Science, Beijing, China).

## 4. Conclusions

In this study, nine compounds **1**–**9** were isolated from *D. fragrans*. Compound **1** is a new coumarin, named as dryofracoumarin A. Compounds **8** and **9**, *trans*- and *cis*-3-(3,4-dimethoxyphenyl)-4-[(*E*)-3,4-dimethoxystyryl]cyclohex-1-ene, which have been previously isolated from the fresh rhizomes of *Zingiber cassumunar* [28,29], were isolated from the genus for the first time, but their anticancer activities have not been reported yet. 

As great attentions have been paid to the development of novel anticancer molecules from TCMs, the anticancer activities of all isolated compounds were tested by the MTT assay. Compounds **2**, **3**, **8** and **9** showed significantly cytotoxic effects against three human cancer cell lines including A549, MCF7 and HepG2, compounds **1** and **5** against two cancer cell lines (A549 and MCF7), and compound **6** against one cancer cell line (MCF7). Consistent with previous studies, the cytotoxic effects of compounds **2**, **3**, **5**, **6** were demonstrated [30,31,32,33,34] again. Importantly, cytotoxic effects of compounds **1**, **8**, **9** were provided for first time. All these active compounds might be promising lead compounds for the treatment of cancer. 
